# Can Quantum Correlations Lead to Violation of the Second Law of Thermodynamics?

**DOI:** 10.3390/e23050573

**Published:** 2021-05-07

**Authors:** Alexey V. Melkikh

**Affiliations:** Institute of Physics and Technology, Ural Federal University, 620002 Yekaterinburg, Russia; melkikh2014@gmail.com

**Keywords:** Carnot cycle, superluminal communication, thermodynamic equilibrium, entanglement, quantum eraser

## Abstract

Quantum entanglement can cause the efficiency of a heat engine to be greater than the efficiency of the Carnot cycle. However, this does not mean a violation of the second law of thermodynamics, since there is no local equilibrium for pure quantum states, and, in the absence of local equilibrium, thermodynamics cannot be formulated correctly. Von Neumann entropy is not a thermodynamic quantity, although it can characterize the ordering of a system. In the case of the entanglement of the particles of the system with the environment, the concept of an isolated system should be refined. In any case, quantum correlations cannot lead to a violation of the second law of thermodynamics in any of its formulations. This article is devoted to a technical discussion of the expected results on the role of quantum entanglement in thermodynamics.

## 1. Introduction

Quantum correlations can affect the state of distant particles, leading to a change in their behavior. To what extent can accounting for quantum correlations affect the formulation of thermodynamics in general and the second law, in particular?

One of the formulations of the second law of thermodynamics is associated with the Carnot cycle. Despite the fact that the Carnot cycle was proposed approximately 200 years ago, at present, investigations regarding it continue (see, for example, [1,2,3,4,5]). A consequence of Carnot’s theorem is that the efficiency of any heat engine cannot exceed the efficiency of an ideal heat engine for the same refrigerator and heater temperatures. This conclusion can be considered one of the formulations of the second law of thermodynamics.

However, in recent decades, ideas have appeared that take into account that quantum effects can, in one way or another, affect both the efficiency of the heat engine and the formulation of the second law of thermodynamics as a whole. This can be especially important for small machines consisting of a small number of particles. Consideration of the quantum effects of small machines has led to the creation of a separate area of study, quantum thermodynamics [6,7]. One of the main questions of quantum thermodynamics can be formulated as follows (see, for example, [8]): can coherence between two internal states of the energy of a machine increase its efficiency?

The question of the limitations of the second law is one of the most fundamental and most important in physics. This issue is of great practical importance for nanomachines. Techniques used in processing quantum information could prove useful for squeezing extra energy out of miniature engines, for instance. These lessons could help scientists build nanomachines that harvest heat and use it to deliver medicine inside the body, or help reduce energy loss in tiny components of traditional computers.

Another important aspect of the application of the second law of thermodynamics to quantum systems is that entangled particles can interact with each other outside of an isolated system. Could the presence of entangled particles in a system affect the formulation of the second law?

This paper is devoted to a technical discussion supporting expected results about the role of von Neumann entropy and system-environment quantum entanglement in the context of non-equilibrium thermodynamics.

## 2. Quantum Carnot Cycle and the Second Law

Several studies [9,10,11] have shown that the internal coherence created during machine operation can affect the measurable thermodynamic properties of the machine and its efficiency.

In the work of Allahverdyan and Nieuwenhuizen [12], it was shown that, in the case of entanglement of a particle with a thermostat, work can be extracted from the thermostat in a cyclic process of changing a certain parameter characterizing the thermostat. The authors point out that such an apparent violation of the second law of thermodynamics is a consequence of the quantum coherence of the thermostat. It can be added that when we are talking about the entanglement of any particle with a thermostat, then we certainly cannot speak of a thermostat in the thermodynamic sense, since in the presence of entanglement, there is no thermodynamic equilibrium and vice versa.

In quantum thermodynamics, cyclic (discrete and continuous) transformations of energy in the quantum regime have been constructed [6]. Moreover, within the framework of quantum information theory, nonthermal states can be used as thermodynamic resources.

In their work, Klatzow and co-authors [8] considered a three-level system as a heat engine. Such a system, as a small machine, can produce more power than an equivalent classical system. The authors used an ensemble of nitrogen vacancy centers in diamond to create two types of quantum heat engines.

The Carnot quantum cycle and other quantum cycles are considered in a number of works [11]. Let us clarify that we are talking not about the use of quantum statistics (for example, in the form of the Fermi or Bose distribution) [13,14] but, rather, about pure quantum states.

Could quantum coherence allow us to build a machine that is more efficient than the Carnot cycle? Could the second law of thermodynamics be thereby violated?

Note that the very formulation of the Carnot cycle, as well as thermodynamics as a whole, is based on the postulate of thermodynamic equilibrium. The statement that there is thermodynamic equilibrium in the system with thermodynamic parameters characterizing it is sometimes called the zeroth law of thermodynamics. The thermal equilibrium postulate leads to the definition of temperature [15] (Definition 1):

*If the system is in a state of thermodynamic equilibrium, then the energy derivative of entropy is the same for all its parts, i.e., constant along the entire system. The inverse of the derivative of the entropy of a body S with respect to its energy E is called the absolute temperature, T:*

(1) $$\frac{1}{T}=\frac{dS}{dE}\;.$$

The temperatures of bodies in equilibrium with each other are the same; *T*_1_ = *T*_2_.

Thus, the definition of temperature is, in any case, related to the determination of thermodynamic equilibrium. The definition of pressure:

(2) $$p=-{\left(\frac{dE}{dV}\right)}_{S}$$

is also associated with the presence of thermodynamic equilibrium, since it is a consequence of the thermodynamic relation:

(3) dE=TdS−pdV

Only in the presence of local thermodynamic equilibrium are all quantities in the last equality defined. Note that the definition of pressure assumes that it is a scalar quantity, i.e., the pressure forces are directed isotropically (Pascal’s law). If this is not the case, then the concept of pressure cannot be introduced.

The features of the zeroth law of thermodynamics within the framework of special relativity are discussed in [16].

Thus, for many macrosystems, it can be argued that they are in a state of thermodynamic equilibrium (local or global), and thermodynamic parameters can be used to describe them.

Now let some part or subsystem of the system be described by a wave function, i.e., it is in a pure quantum state.

Obviously, in this case, the zeroth law of thermodynamics is not fulfilled. If this system is now part of a machine, then it is clear that there can be no question of the Carnot cycle in this case, which can only be formulated in terms of thermodynamics. That is, the thermodynamic equilibrium state cannot simultaneously coexist with the pure state of the system.

In a pure quantum state, a system is known to be described by a wave function. Probabilities are formed only as a result of the transition to the mixed state. With subsequent thermalization, the system can reach equilibrium. The processes of decoherence accompanied by the creation or annihilation of particles destroy entanglement. The same processes thermalize the system. Thus, in equilibrium, any entanglement in the system cannot exist.

Note that in quantum systems, there is no natural analog of the Carnot cycle, but there are criteria for the efficiency of energy conversion. For example, the efficiency of transport, according to [17], has the following form:

(4) $$\eta =2\underset{m}{\sum }k_{m}\int_{0}^{\infty }dtm\langle |\rho \left(t\right)|m\rangle$$

This efficiency, being a dimensionless quantity, does not contain thermodynamic parameters and is not associated with any limitations of the Carnot cycle.

In this case, can we say that a quantum machine can have an efficiency greater than the efficiency of the Carnot cycle? Of course, yes. However, this does not say anything about the violation of Carnot’s theorem or about the violation of the second law of thermodynamics since they are formulated for another case—thermodynamic equilibrium.

Thus, we can describe the system in terms of thermodynamics, but this is possible only if the system is in local equilibrium. In this case, no part of the system can be in a pure state. Therefore, we cannot speak about quantum information and entanglement between quantum particles.

Alternatively, we can describe the system based on the quantum mechanics of pure states (wave functions). Entanglement and other quantum effects can be present in this system. Then, the efficiency of a machine built on such effects can be arbitrarily high (if there are no restrictions on the particles that make up such a machine). In any case, it can be greater than the efficiency of the Carnot cycle. However, we are not talking about thermodynamics at all since its zeroth law is violated. If the zeroth law is violated, the rest of the laws of thermodynamics cannot be correctly formulated. In this case, it makes no sense to compare the efficiency of such a machine with the efficiency of the Carnot cycle, since these two systems are in different conditions.

The foregoing applies, not only to the Carnot cycle, but also to thermodynamics in general. If a part of a system is described on the basis of quantum mechanics of pure states, then this particular part is not described by thermodynamics, and vice versa. It makes no sense to say that quantum mechanics violates thermodynamics in some way. These sciences just do not work together.

The impossibility of the existence of a perpetuum mobile of the second kind, which can act indefinitely and convert all the heat received from other bodies into work, is one of the formulations of the second law of thermodynamics. For example, Nikulov [18] proposed as such a perpetuum mobile, the existence of a direct current with nonzero resistance due to the quantization of the angular momentum.

However, this is not a violation of the second law, since perpetual motion itself (for example, the motion of electrons in atoms) is the basis of quantum mechanics. Such movement has nothing to do with thermodynamics, since there is no thermodynamic equilibrium—the basis of thermodynamics.

## 3. Quantum Delayed Eraser, Superluminal Communication and the Second Law

Consider an experiment with a delayed quantum eraser [19]. The essence of this experiment is that there are two entangled photons, one of which is a signal. The signal photon is directed to two slits or an interferometer, while the other is directed to polarizers and detectors. The delayed version consists of detecting the first photon before the second. According to the authors [19], information about one or both paths can be erased using the second photon even after the first is registered.

At the same time, it is noted by Kastner [20] that in this experiment, the information is not erased but is only transferred to another subsystem. An analogy is drawn between the experiment of a quantum eraser and an EPR experiment. Slits A or B completely correspond to the spins along the *z*-axis and *x*-axis. Therefore, we can say that two photons in the experiment of a quantum eraser are in the EPR state.

In a quantum eraser experiment, the experimenter observes which slit each photon passes through and demonstrates that after that, the interference pattern is destroyed. This stage shows that the presence of detectors causes the destruction of the interference pattern. The result of “erasing” information about the chosen photon path is restoration of the interference pattern.

The quantum eraser experiment carried out in [19,21] is a variation of the classical Young experiment on two slits, which establishes that a photon cannot interfere with itself when the experimenter tries to determine which slit the photon has passed through. When the photon flux is subjected to such an observation, the interference stripes characteristic of Young’s experiment are not observed. The quantum eraser experiment is capable of creating situations in which a photon that has been “marked” to determine which slit it has passed through can subsequently be “cleaned” of such marking. This is possible because subsequent measurement of the conjugate quantity makes the original quantity undefined. A “marked” photon cannot interfere with itself and will not generate fringes, but a photon that has been “marked” and then “cleaned” can subsequently interfere with itself and will contribute to the generation of interference stripes similar to those obtained in Jung’s experiment.

Quantum erasure experiments show that entangled systems can influence each other even if they are at a considerable distance and do not interact directly.

In particular, with respect to the second law of thermodynamics, a consequence of the entanglement of a part of a quantum system with the environment is that it is not obvious when the quantum system is isolated. For a classical system, if external bodies are far from the system, then this system can be considered isolated with good accuracy, since their contribution to its energy will be small. For a quantum system, even if the outer bodies are far away and their contribution to the energy is small, one must take into account their possible entanglement with the system. *A quantum system can be considered isolated if it does not exchange energy with the environment and is not entangled with it* (Definition 2). However, an alternative definition is also possible, since entanglement is not related to energy transfer.

Consider a thought experiment with an isolated quantum system based on a quantum eraser experiment. Let us now consider not two but *N* entangled signal photons, which can be considered an isolated system. Let us conduct a similar experiment on erasing information with them. For these photons, the “erasure” of information will lead to the fact that they will again be in a pure state, i.e., their von Neumann entropy will change. At first glance, this behavior of photons contradicts the second law of thermodynamics, since in an isolated system, the state of particles is ordered. However, there is no equilibrium in such a system; therefore, it is impossible to talk about the violation of the second law.

It is possible to verify that the system is energetically isolated by checking the total energy storage. In entangled systems, as well as in nonentangled systems, energy should definitely be conserved.

An alternative is a more precise definition of an isolated system in quantum mechanics: a system is isolated if it does not interact with the surrounding bodies and is not entangled with them. The disadvantage of this definition is that entanglement depends on the prehistory of interactions between particles.

The violation of the second law of thermodynamics has been proposed as an argument against the spread of information faster than the speed of light [22]. According to the author, if there is no interaction between two entangled particles (for example, in the EPR experiment), but when one is measured, the state of the other also changes, then this corresponds to an additional decrease in the entropy of an isolated system. Since such processes are prohibited by the second law of thermodynamics, this is precisely what is considered a prohibition of superluminal communication. However, this argument implicitly implies that there are thermodynamic parameters in the system, such as entropy and temperature. As discussed above, such parameters can be introduced only in the presence of thermodynamic equilibrium.

Thus, the second law of thermodynamics cannot serve as a ban on superluminal communication and vice versa.

Quantum correlations between particles can be considered in a more general context. According to Braunstein and Pati [23], quantum information cannot be completely hidden in quantum correlations. The authors considered an arbitrary system (mixed or entangled with some external system), which is encoded in some larger Hilbert space using some unitary process. Assuming that this encoding process completely hides information about the state of a certain subsystem of this Hilbert space (the state of the subsystem does not depend in any way on the hidden state), the authors proved that the hidden information is completely encoded in the remaining Hilbert space. In addition, the authors proved that it is possible to hide quantum information only by transferring it to another subsystem.

The no-hiding theorem is applicable to arbitrary information erasure processes, including thermalization. Hiding quantum information is equivalent to erasing it, and completely hiding it means complete thermalization. In the case of incomplete hiding, the quantum information will be stored somewhere in the environment of the system.

In relation to the second law of thermodynamics, the following can be a consequence of the no-hiding theorem. Thermalization of one system always occurs at the expense of some other. In thermodynamics, such a system is a thermostat. However, a thermostat is by definition in equilibrium and cannot contain quantum correlations. If the thermostat contains quantum correlations, then, as noted above, strictly speaking, we are no longer talking about a thermostat.

In this regard, it should be noted that, at the microscopic level, entanglement exists in all systems. Entanglement between two specific particles usually does not exist for a long time, it is destroyed due to the processes of decoherence associated with the creation (annihilation) of particles. This microscopic entanglement does not contradict thermodynamics in any way, since in thermodynamics average values are considered, and fluctuations are neglected. Macroscopic entanglement, which is discussed in this article, is incompatible with thermodynamics.

In [24] it is argued that the main postulate of statistical mechanics should be replaced by the general canonical principle. The key element in proving a new principle is the quantum entanglement between the system and its environment. However, it remains unclear to what extent the conclusions made by the authors are applicable to specific quantum systems, such as, for example, a superfluid liquid. In many cases, thermodynamics and quantum mechanics give distinctly different predictions.

On the other hand, the authors do not claim that their conclusions refer to the Boltzmann distribution and do not introduce thermodynamic parameters. Thus, the foundations of statistical physics and the applicability of quantum mechanics to thermodynamic systems are different problems.

## 4. Von Neumann Entropy Is Not a Thermodynamic Quantity

Let us show, however, that the violation of the second law in the above thought experiment is only apparent since the von Neumann entropy is not a thermodynamic quantity. The formula for the von Neumann entropy has the form

(5) $$S_{vN}=-Tr\left(\rho \ln\rho \right)$$

where *ρ* is the density matrix. The density matrix contains diagonal and off-diagonal elements. Only diagonal elements correspond to probabilities. If the matrix contains only diagonal elements, the von Neumann entropy corresponds to the classical entropy.

However, can von Neumann entropy be considered a thermodynamic quantity? Is it possible to draw conclusions about the second law of thermodynamics on the basis of its change?

According to Landau and Lifshitz [15], for the entropy the following relation holds:

(6) $$S=-\underset{n}{\sum }w_{n}\ln w_{n}$$

On the other hand, Boltzmann’s formula (6) for a physical macrosystem was derived under the condition of thermodynamic equilibrium. Indeed, the expression for entropy can be obtained as follows (see, for example, [25]) from the first law of thermodynamics. Writing the first law as

(7) dU=dQ−dA

and substituting work into this expression

(8) $$dA=\int \left(\overset{n}{\underset{i=1}{\sum }}\frac{\partial H\left(X,a\right)}{\partial a_{i}}da_{i}\right)f_{N}\left(X,a,T\right)dX$$

and internal energy

(9) $$U\left(a,T\right)=\int H\left(X,a\right)f_{N}\left(X,a,T\right)dX$$

We obtain expression for the heat differential

(10) $$dQ=\int H\left(X,a\right)d_{a,T}\left(f_{N}\left(X,a,T\right)\right)dX$$

Here *a* is the set of external parameters, *H*(*X*,*a*) is the Hamiltonian of the system and *f_N_* (*X*, *a*, *T*) is the distribution function for *N* particles. Using the canonical Gibbs distribution, we obtain the final expression for the entropy:

(11) $$S\left(a,T\right)=-k_{Б}\int \ln f_{N}\left(X,a,T\right)f_{N}\left(X,a,T\right)dX+S_{0}$$

Formula (11) or the expression for the discrete case

(12) $$S\left[n\right]=-k_{Б}\underset{n}{\sum }f_{n}\ln f_{n}$$

is a measure of the disorder of the system. Note that the use of the canonical Gibbs distribution means that the system is in equilibrium and is described by temperature. Only on this basis can the desired formula for entropy be obtained. Thus, the formula for entropy (6), widely used for various physical systems, assumes the presence of thermodynamic equilibrium.

In systems with pure quantum subsystems, in the general case, equilibrium is absent, which does not allow the introduction of thermodynamic entropy for such systems. That is, it cannot be written that:

(13) $$dQ=TdS_{vN},$$

because the temperature in a system that is in a pure quantum state is not determined.

Thus, it should be emphasized that the formation of probabilities (rather than wave functions) is not enough to apply formula (6) and the formulation of thermodynamics. It is precisely the local thermodynamic equilibrium that is needed. Only such entropy can correspond to thermodynamics, and only on this basis can other thermodynamic relations be used.

That is, the von Neumann entropy is not a thermodynamic quantity, although it can characterize the ordering of the system in many cases.

## 5. Conclusions

Although the erasure of quantum information, as well as the measurement of the state of one of the particles in the EPR pair, can lead to a change in the von Neumann entropy of an isolated system, which is entangled with some other distant system, this will not lead to a violation of the second law of thermodynamics, since it does not relate to thermodynamic quantities. When the system is in local equilibrium, it is possible to introduce thermodynamic quantities (including thermodynamic entropy), but entanglement, EPR paradox and the erasure of quantum information cannot take place. A quantum machine cannot have an efficiency greater than that of a Carnot cycle since entanglement cannot exist simultaneously with the equilibrium state for which Carnot’s theorem was formulated. Quantum effects cannot violate the laws of thermodynamics; however, their presence will clarify their formulations in relation to quantum systems and impose some restrictions on the application of thermodynamics.

The question of a possible violation of the second law of thermodynamics is of practical importance for the creation of nanoengines consisting, for example, of one atom (see, for example, [26]). The second law of thermodynamics is not violated during the operation of such engines, however, for nanoengines there may be other limitations associated with quantum mechanics.

## Data Availability

No new data were created or analyzed in this study. Data sharing is not applicable to this article.
